# Methodology of Maternal and Child Health Populational Surveys: A Statewide Cross-sectional Time Series Carried Out in Ceará, Brazil, from 1987 to 2017, with Pooled Data Analysis for Child Stunting

**DOI:** 10.5334/aogh.2299

**Published:** 2019-03-04

**Authors:** Luciano Lima Correia, Hermano Alexandre Lima Rocha, Sabrina Gabriele Maia Oliveira Rocha, Lucas Silveira do Nascimento, Anamaria Cavalcante e Silva, Jocileide Sales Campos, Álvaro Jorge Madeiro Leite

**Affiliations:** 1 Department of Community Health, Federal University of Ceará, Rua Prof. Costa Mendes, Fortaleza, CE, BR; 2 Christus Univesity Center, R. João Adolfo Gurgel, Cocó, Fortaleza, CE, BR

## Abstract

**Background:**

Cross-sectional studies are fundamental studies in the practice of epidemiological science. This article aims to present in detail the methodology for conducting a series of cross-sectional studies, as well as the analysis of data through pooled data.

**Methods:**

The series of studies are population cross-sectional studies, with statewide coverage, searching for representative sample of reproductive aged women and pre-school children in Ceará, Brazil. The sampling plan followed simple random, stratified, systematic and by conglomerates, in sequence. About 300 variables were collected. For each of the individual studies, multivariate data analysis was used to verify associations between dependent variables. For all the studies together, techniques used were trend chi-squared and pooled data analysis using linear mixed modeling procedures.

**Results:**

There were 6 studies in sequence, for 30 years. Among other findings, the variables income, maternal education and breastfeeding time proved to be associated with the reduction of malnutrition in children considering all the period (p values 0.013, 0.033 and 0.037, respectively).

**Conclusions:**

Cross-sectional studies can be replicated at regular time series following the methodology exposed in this, even for locations with limited resources, ensuring adequate management of decisions of using federal funding aimed at achieving targeted programs to maximize the results obtained with the public resource available.

## Background

Cross-sectional studies are essential kind of studies to epidemiological practice [1]. Although they do not present temporality, which affects their strength for defining causal relationships, they are very important for the situational diagnosis of a population, allowing the creation of effective health management strategies, being used by several government agencies for this purpose [2,3,4,5]. Since 2009, UNICEF has encouraged the implementation of the Multiple Indicator Cluster Survey, in order to fill the gaps on data of the situation of children and women monitoring [6].

In Brazil, some states have developed cross-sectional studies of statewide scope for the evaluation of maternal and child health, of which the state of Ceará was one of the first to do so in 1987 [7]. In this first survey, the Maternal of Ceará (PESMIC) identified, for example, the high infant mortality rate (IMR) due to diarrhea, from which policy strategies were proposed to reduce IMR, such as the dissemination of oral rehydration therapy [8,9]. The recognition of the relevance and importance of this study in the state of Ceará stimulated its continuity, and the study has been done 6 times more, thus making one of the few transversal series of maternal and child health. Since then, its information has been used to understand the determinants of child malnutrition and to assist in combating measles outbreaks, for example [10,11]. Through these studies, the important impact of income and other social determinants on child malnutrition in Brazil was identified, allowing government action [12].

The transversal series and their analysis through pooled data is well studied and known in the area of social sciences, such as economics and politics, however there are not many studies that have used these methodologies in health area [13,14].

This article aims to present in detail the methodology for conducting series of cross-sectional studies with statewide coverage, as well as one of the possible recommended forms for analysis of results through pooled data, in order to contribute for the international efforts of health information improvement and better intervention strategies. As an example of the application of this methodology, we also aim to identify determinants of malnutrition.

## Methods

The methodology used in the series of PESMIC studies is presented according to three main elements, which are specified as below:

- Study design: Study population; Sampling plan; Main variables; Questionnaires; Plan of data analysis and Pooled data analysis techniques; Ethical requirements.
- Data collection procedures: Logistics of the field work; Materials and instruments; Quality control; Financing of investigations.
- Results of the study sample: Interviews performed in each study; Socioeconomic characteristics of the sample; Analysis of determinants of malnutrition in the period.

The methodology used in the first five series of PESMICs was strictly the same, with the same population groups, changing only in the sixth sampling, as will be explained next. In all the researches, the basic socioeconomic and health indicators were investigated. In each survey, however, the main focus of the study ranged from infant mortality in the first study (1986), to maternal health in the second (1990), reproductive health in the third (1994), family health in the fourth (2001), maternal and child nutrition in the fifth (2007) and child development in the sixth PESMIC (2017).

### Study design

The series of PESMIC are cross-sectional studies based on population surveys, with statewide coverage, searching a representative sample of reproductive aged women and pre-school children in the state of Ceará.

#### Sampling

The sample size of 8,000 households was initially established in the first study (1987) to estimate the infant mortality rate in the state. This high number of households was necessary in order to detect a sufficient number of infant deaths to calculate the rate.

Considering the fecundity of the time, it was expected to obtain about 4,000 children with that sample, which would provide an absolute precision of 1, for an estimated event frequency of 10% (infant mortality at that time), at a level of significance of 5%. In subsequent studies, however, the reported number of deaths of children who have died in the previous 12 months declined gradually, rendering a reliable estimate of infant mortality unfeasible. In addition, the number of children declined gradually over the years, due to the fall in fertility rate in Ceará, reaching in the survey of 2007 only 1,500 children under 3 years old identified in 8,000 households.

As a strategy to increase the number of children in the sample, in the last PESMIC 2017, only households with children were studied, increasing the age group from 0 to 3 years of age to 0 to 6 years of age. Consequently, it was possible to reduce the number of households visited, from 8,000 to 3,200 households, with a minimum loss of sample power (absolute precision of 1.04). In all studies between 1987 and 2017, all households visited has provided women for the study. In order to guarantee the representativeness of the studied population, the selection of municipalities, sectors and households was performed in a random manner, obeying a multi-stage sampling process.

The sampling plan has followed the steps below:

- Stratified sampling in order to consider the population proportion of the capital and the countryside of the state, as well as by health region, geographical regions that holds similar health characteristics;
- Systematic sampling for the selection of the municipalities to be surveyed, with 40 municipalities;
- Sampling by clusters, choosing the households within each municipality. In this last stage, the census tracts of the Brazilian Institute of Geography and Statistics (IBGE) were used (geographical areas of variable extensions, but with a uniform population of 300 families) to localize the conglomerates of 20 houses.

A list of all municipalities with their respective populations, sequenced by health region that served as strata, was initially created to ensure an adequate geographic distribution of the sample. After this, the first individual that would be part of the study was randomly drawn, with a random draw of a number between 1 and 8,452,381 (the total population of the state). For that, we used the site *random.org*, which generates its randomization based on atmospheric data. After the draw of the first individual, the size of each step in the systematic sampling was defined, dividing the population by the number of 40 desired municipalities, and obtaining in the case the number 211,309. By this process, a large municipality, to have its population weight respected in the sample, could be raffled more than once. In fact, the State Capital, Fortaleza, with 2,300,000 inhabitants, was selected ten times, and one municipality in the countryside (Caucaia) was selected twice. Thus, at the end of the systematic sampling process, 29 municipalities composed the final sample, instead of 40, as initially established.

The Excel spreadsheet used for the development of this strategy is available as supplementary material, with five sampling examples using this technique.

**Figure 1:**
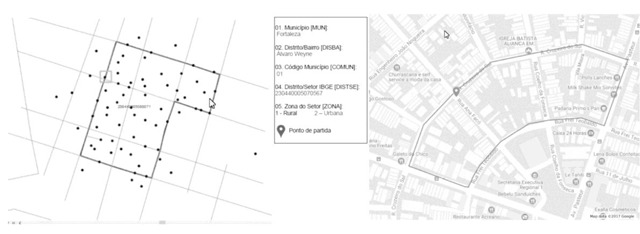
Right: Example of possible randomly generated starting points. Left: Census sector drawn, already with map overlap, delimited by the line, with random starting point signaled by the arrow.

In each municipality, four census tracts in urban and rural areas were selected. These sectors were drawn by simple random selection. Once the sector was drawn, and its respective map obtained from the IBGE, the location of the conglomerate of 20 houses to be researched within the sector was determined by lot, using ArcGis software in version 10.1. The first domicile to be visited was also determined by lot. The resulting *kml file has been exported to mymaps. The choice of this function was due to the greater precision of the street and the greater availability of information of points that can be used as reference in the field, such as trades, schools, churches, etc. (Figure 1).

### Variables

The questions researched on children, women and their families evolved as new interest topics were included in the global, national or local agenda of maternal and child health in the consecutive PESMICs (Table 1). The basic variables, however, remained in all six questionnaires, allowing for comparative analyzes throughout the series. The following variables apply to this characteristic:

- Maternal literacy: ability to read and understand a simple message on health education;
- Family income: the total monthly income earned by all family members, expressed in minimum wages;
- Exclusive breastfeeding: strict use of breast milk, without water or teas;
- Low birth weight: birth weight below 2,500 g;
- Low weight for age: child’s weight for age below –2 standard deviations;
- Growth delay: height of child for age below –2 standard deviations;
- Emaciation: child’s weight for height below –2 standard deviations.

**Table 1:** Evolution of the content of the questionnaires of the PESMIC’s, 1987–2017.

Year	1987	1990	1994	2001	2007	2017

Main focus	Child health	Women’s health	Reproductive health	Family health	Maternal and child nutrition	Child development

Kids	Age and sex	Age and sex	Age and sex	Age and sex	Age and sex	Age and sex
	Birth weight	Birth weight	Birth weight	Birth weight	Birth weight	Birth weight
	Power supply	Power supply	Power supply	Power supply	Power supply	Power supply
	Vaccination	Vaccination	Vaccination	Vaccination	Vaccination	Vaccination
	Morbidity: diarrhea, TRO and IRAs	Morbidity: diarrhea, TRO and IRAs	Morbidity: diarrhea, TRO and IRAs	Morbidity: diarrhea, TRO and IRAs	Morbidity: diarrhea, TRO and IRAs	Morbidity: diarrhea, TRO and IRAs
	Attend day care	Attend day care	Attend day care	Attend day care	Attend day care	Attend day care
	Medical consultations	Medical consultations	Medical consultations	Medical consultations	Medical consultations	Medical consultations
	Hospitalization	Hospitalization	Hospitalization	Hospitalization	Hospitalization	Hospitalization
	Health agent visits	Health agent visits	Health agent visits	Health agent visits	Health agent visits	Health agent visits
	Nutritional evaluation	Nutritional evaluation	Nutritional evaluation	Nutritional evaluation	Nutritional evaluation	Nutritional evaluation
					Accidents at home	Accidents at home
						Evaluation of autism
						Early stimulation
						Child development measured by ASQ3

Women	Age	Age	Age	Age	Age	Age
		Schooling	Schooling	Schooling	Schooling	Schooling
		Prenatal care	Prenatal care	Prenatal care	Prenatal care	Prenatal care
		Childbirth assistance	Childbirth assistance	Childbirth assistance	Childbirth assistance	Childbirth assistance
		Work outside the home	Work outside the home	Work outside the home	Work outside the home	Work outside the home
		Reproductive history	Reproductive history	Reproductive history	Reproductive history	Reproductive history
		Cancer prevention	Cancer prevention	Cancer prevention	Cancer prevention	Cancer prevention
		Family planning	Family planning	Family planning	Family planning	Family planning
			Surgical sterilization	Surgical sterilization	Surgical sterilization	Surgical sterilization
			Gynecological morbidity	Gynecological morbidity	Gynecological morbidity	Gynecological morbidity
			Medical consultations	Medical consultations	Medical consultations	Medical consultations
			Health agent visits	Health agent visits	Health agent visits	Health agent visits
				STDs and AIDS	STDs and AIDS	STDs and AIDS
					Smoking	Smoking
					Hypertension and Diabetes	Hypertension and Diabetes
					Nutritional evaluation	Nutritional evaluation
						Other forms of morbidity
						Maternal Depression

Family	Number of people	Number of people	Number of people	Number of people	Number of people	Number of people
				Head of the family	Head of the family	Head of the family
				Education Chief family	Education Chief family	Education Chief family
	Family income	Family income	Family income	Family income	Family income	Family income
	Water and sanitation	Water and sanitation	Water and sanitation	Water and sanitation	Water and sanitation	Water and sanitation
			Access to the Family Health Program (PSF)	Access to the Family Health Program (PSF)	Access to the Family Health Program (PSF)	Access to the Family Health Program (PSF)
			Health insurance	Health insurance	Health insurance	Health insurance
					Food security	Food security
					Cash transfer Program	Cash transfer Program
						Social adversities
						Domestic violence
						Presence of toxic stress

The data had double entry in computer, the two databases being validated for errors of typing. In the data processing, the Epi Info 6.04 (CDC/WHO) software was used in PESMIC’s of 1987, 1990 and 1994 and Epi Info 2000 in 2001, 2007 and 2017.

The nutritional analysis of the children generated weight-for-age, stature by age and weight by stature scores. Standard deviations were calculated for these anthropometric indices in comparison to those of an ‘international reference population’, as defined by the US National Center for Health Statistics (NCHS). In the data analysis of the last two PESMICs, the international growth reference developed by the World Health Organization was used. The following indicators of nutritional disorders were produced with the anthropometric measurements of the children:

- Acute malnutrition: Weight per Age (W/A) < –2 Z scores;
- Obesity: Weight per Age (W/A) > 2 Z scores;
- Nutritional dwarfism: Height per Age (H/A) < –2 Z scores;
- Wasting: Weight by Height (W/H) < –2 Z scores.

The anthropometric measurements of the women allowed the calculation of the Body Mass Index (BMI), which correlates weight and height, according to gender and age, and to parameters recommended by WHO [15]. The deficits and excesses found in these anthropometric indices in relation to the reference population, considered ‘normal’, has evidenced in this study the nutritional disorders studied, with their respective classification criteria.

BMI classification (kg/m^2^):

- Low weight < 18.5
- Normal weight 18.5 to 24.9
- Overweight ≥ 25
- Pre-obese 25.0 to 29.9
- Obese I 30.0 to 34.9
- Obese II 35.0 to 39.9
- Obese III ≥ 40.0.

#### Data collection procedures

Field researchers worked in pairs, each pair covering a sector (conglomeration of 20 houses) per day. The fieldwork was scheduled to be executed within 45 calendar days, or 34 business days, considering the non-occurrence of unforeseen events.

In each household, all the resident women and children in the age groups of research had their anthropometric measurements recomputed and the respective questionnaires applied.

After the completion of data collection in the first home, the field work progressed, following specific rules: a) the researcher visited the houses following the clockwise; b) Commercial establishments and homes without residents were not included in the conglomeration of 20 houses, these being replaced by other neighbors; c) in the case of missing families, up to three callbacks were performed in an attempt to obtain the data from home.

Information was collected using three different questionnaires. The first recorded information of each home included in the sample; the second collected information for all women from 10 to 49 years of age residing in households visited, so while women who had been pregnant answered the complete questionnaire, those still without reproductive experience answered only basic epidemiological questions; the third questionnaire was about child health, and it was applied to all responsible people for children under three years of age living in households visited.

For the assessment of nutritional status, it was carried out the verification of the weight, height, and waist and hip circumference in both women and children of the age groups surveyed. The characteristics of the equipment used in anthropometry are presented below.

The equipment was calibrated on a regular basis using standardized measures, earlier in the day and every 25 measurements [16].

Inter-interviewers’ variability in the calibration procedures was monitored by repeated measurements. Supervisors accompanied the interviewers on their first sessions.

During the field work a subsample of 10% of children and women (2 households in each conglomeration of 20) were measured again by supervisors, on a blind basis, as a mean of quality control of the work of interviewers.

The questionnaires were daily reviewed by supervisors in order to identify any mistakes and fill correctly the data, if possible.

### Funding of surveys

Since the ending of the activities of the PESMIC 2007, IE, collection, database construction, analysis, reporting to funding agencies, publication of the results, and communication to managers in sessions, the researchers have started to search for resources in order to finance the following study. It was built a new project with schedule and budget, as well as the group of people and entities that would give support to the study was set.

### Statistical analysis

In the PESMICs of 2007 and 2017, the Anthro program was used to analyze the anthropometric data and produce the Z scores of child nutrition, replacing the EpiNut program used until then.

The feeding of the data generated three distinct databases, one for home, one for women and one for children. It may be more than one woman per household and more than one child per woman. For this purpose, we used the merge command, using a key variable, where the two banks provided cases for addition of variables with matching cases.

For each of the individual studies, multivariate data analysis was used to verify associations between dependent variables, e.g., nutritional, events and independent variables, as the reason of association prevalence by Cox regression, with the backward modeling strategy [17], as published in articles on other sites using the data from these studies [10,11].

For all the studies together, the techniques used were trend chi-squared and pooled data analysis, using linear mixed modeling procedures whereas territorial units of Ceará, regions with completely different health indicators and the year of the study, were used as fixed factors. These analyses are presented in this study, taking as an example the determination of chronic child malnutrition (height for age) by the variables income (in minimum wages to minimize inflationary variation), nursing time, birth weight and maternal schooling, after natural logarithmic conversion. As the malnutrition is measured in Z scores, which shows a negative result, a constant was added to the original variable for conversion. Adjustment measures were estimated to allow choosing the best model, that was less impacted by this adjustment strategy [18]. Also, there are presented the Chi-square test for trend.

Using the software SPSS version 23, IBM Inc., to carry out the analysis, it was considered 0.05 as the level of significance.

#### Ethics

In all PESMICs, terms of informed consent have been applied to the women and children through their mothers. The protocols of the studies were submitted and approved by the research ethics committees.

## Results

The databases generated by PESMIC offer information on a total of 39,822 families, 47,506 women of childbearing age and 13,049 children under three years of age (Table 2). Only a small number of 178 families refused to cooperate with the research, and all these cases have occurred in the two most recent surveys. It’s worth pointing out that the absence of family is not considered a refusal, and in these cases the residence was replaced by the following.

**Table 2:** Distribution of sample elements of five PESMICSs: households, people resident, women of childbearing age and preschool kids. Ceará, Brazil, 1987 to 2007.

Years	Households	Residents	Women of 10–49 years	Children 0–3 years	Children from 4–6 years

1987	8,000	39,653	10,743	4,513	–
1990	8,000	31,204	8,476	2,861	–
1994	8,000	35,935	9,710	2,461	–
2001	7,833	35,535	9,754	1,681	–
2007	7,989	34,805	8,825	1,533	–
2017	3,200	13,249	3,752	2,153	1,413
Total	43,022	190,381	51,260	15,202	1,413

The number of women available for interviews in the homes has fluctuated a bit of an investigation to the other, keeping around 9,000, since in each residence were included all eligible women. With respect to children, however, the decline was sharp, going from 4,513 children younger than three years of age found in households in 1987, to only 1,533 in 2007, representing a reduction of 66%.

Important changes have occurred in the socioeconomic and demographic indicators of the population studied in this 30-year period, as can be seen in Table 3. About half of the families persevered receiving up to one minimum salary per month during the period of 30 years. However, despite the economic indicator have not improved, other indicators relevant to the improvement of maternal and child health, such as access to drinking water and female education, have evolved significantly, with increases of 136 and 65%, respectively, in two decades. The reduction in the size of families was another phenomenon observed during this period, which presents a strong impact on the economy and on the family health, since it potentially represents a little more resources and care for the children. (Table 3)

**Table 3:** Socioeconomic and demographic characterization families’ participants of PESMICs. Ceará, Brazil, 1987 to 2007.

Indicator	Years						% change**	P value**

	1987	1990	1994	2001	2007	2017		

% of households with monthly income ≤ 1 minimum wage	52.5	46.5	47.3	49.4	58.1	56.1	+10.7	<0.001
% of households with potable water availability^1^	25.3	–	25.8	67.8	77.0	84.1	+136.4	<0.001
% of females literate	58.4	75.1	93	92.7	96.4	96.7	+65.1	<0.001
% Coverage for measles	48.2	76.3	94.4	94.9	96.7	87.2	+80.9%	<0.001

PESMIC = Maternal and Child Health Survey of Ceará. * Difference between the last and the first measurement. ** Chi-square trend. ^1^ Piped water and/or mineral water.

During the period, it was found the reduction of child malnutrition until 2007, with an increase in 2017, accompanied by increased maternal education, increased time of breastfeeding, birth weight stable and variation in the income situation. The variables income, maternal education and breastfeeding time have proved to be statistically associated (p values 0.013, 0.033 and 0.037, respectively) with the reduction of malnutrition in the multivariate model (Table 4).

**Table 4:** Pooled data analysis of determinant factor of chronic undernutrition over the 30 years.

Model Variables*													P value

Fixed											
Year	1987	1987	1990	1990	1994	1994	2001	2001	2007	2007	2017	2017	
Main city or Country town	Main city	Country town	Main city	Country town	Main city	Country town	Main city	Country town	Main city	Country town	Main city	Country town	
Dependent											
Height for age	2.28	0.73	–0.53	–1.10	–0.71	–1.02	–0.20	–1.03	0.44	0.31	–0.0276	–0.3814	
Independent											
Maternal education	6.02	3.57	6.19	2.85	6.38	3.45	6.66	5.78	6.89	6.46	7.77	7.48	0.037
Time to weaning from breastfeeding	2.54	2.86	3.53	5.00	5.05	6.05	6.21	6.33	6.62	7.75	9.87	9.57	0.033
Birth weight	3,318.83	3,399.82	3,324.35	3,364.51	3,227.86	3,309.09	3,349.34	3,317.78	3,221.77	3,286.98	3,254.03	3,233.08	0.113
Family income	5.49	1.41	4.68	1.74	2.70	1.65	4.94	1.77	2.60	1.21	1.36	1.14	0.013

* Adjustment model measures: Akaike information criteria (AIC) –10,848. Hurvich and Tsai criterion (AICC) –2,848. Bozdogan criterion (CAIC) –8,010. Schwarz’s Bayesian criterion (BIC) –11,010.

## Discussion

This data shows it has been possible to accomplish, with relative success, a series of cross-sectional population-based studies that has been through three decades, monitoring the health and nutritional situation of two population groups of high vulnerability (children under three years of age and women of childbearing age) living in Brazil’s semi-arid region, known as the highest concentration of poverty in the whole country. The pooled data analysis in this study confirms, through data analyzed over 30 years, the relevance of the income, the low birth weight, and the breastfeeding in the nutritional status of children, as yet not identified using this method until now.

The databases of the six studies together provide detailed information about 45,000 families, allowing us to carry out several analyses, including historic tracts, on the evolution of maternal and child health. The interconnection of the three questionnaires, the woman, the child and the domicile, enhances even more the possibilities of analysis.

There are several international examples of the use of this methodology for public health, in Brazil and in other countries, with different degrees of methodologic rigor and number of studies [19,20]. The techniques of sampling, description of variables and statistical analysis used are relevant so that the result is relevant.

It has been possible through the PESMIC’s to register the presence of the phenomenon of epidemiological and nutritional transition in the maternal and child population of Ceará, which have important implications on changing public health policies, food and nutrition for the Brazilian Northeast region. There were stated in two publications of PESMIC the current nutritional situation analysis of women and children, regarding to obesity and malnutrition, which are essential to guide the management of the social and health areas in the formulation of strategies for the tackling both problems [10].

The experience of PESMIC’s has generated a fruitful process of integration of the health services and the University, in which the results of the research have been dynamically interacting in the formulation of public policies for maternal and child health care for the State, resulting in a consistently positive impact on the health status of this population, being internationally recognized [9].

The strategy to keep the same methodology for all studies was fundamental to ensure undisputed comparability within the time series. In fact, every reprint of the entire sequence of study PESMIC is renewed and the historical series win more importance.

The use of transversal series allows to maximize the results and effects caused by each of the individual studies, bringing new findings that may lead to different management strategies.

It is concluded that the cross-sectional studies can be replicated at regular time series following the methodology exposed in this paper, even for locations with limited resources, ensuring adequate managements of decisions by using federal funding aimed at achieving targeted programs to maximize the obtained results with the public resource available, as could be exampled by the determinants of stunting identified (income, maternal education and breastfeeding).
